# Effective microorganisms input efficiently improves the vegetation and microbial community of degraded alpine grassland

**DOI:** 10.3389/fmicb.2023.1330149

**Published:** 2024-01-15

**Authors:** Jinsheng Li, Juping Wei, Xinqing Shao, Xinhui Yan, Kesi Liu

**Affiliations:** ^1^School of Resources and Environment, Anhui Agricultural University, Hefei, Anhui, China; ^2^College of Grassland Science and Technology, China Agricultural University, Beijing, China; ^3^Key Laboratory of Restoration Ecology of Cold Area in Qinghai Province, Northwest Institute of Plateau Biology, Chinese Academy of Sciences, Xining, China

**Keywords:** effective microorganism, alpine grassland, soil nutrient, arbuscular mycorrhizal fungi, restore effect

## Abstract

Soil beneficial microorganism deficiency in the degraded grasslands have emerged as the major factors negatively impacting soil quality and vegetation productivity. EM (effective microorganisms) has been regarded as a good ameliorant in improving microbial communities and restoring degraded soil of agricultural systems. However, knowledge was inadequate regarding the effects of adding EM on the degraded alpine grassland. Four levels of EM addition (0, 150, 200, 250 mL m^–2^) were conducted to investigate the effects of EM addition on soil properties and microorganisms of degraded alpine grassland. The addition of EM increased aboveground biomass, soil organic carbon, total nitrogen, available phosphorus, and microbial biomass, but decreased soil electric conductivity. Meanwhile, the relative biomasses of gram-negative bacteria decreased, while the ectomycorrhizal fungi and arbuscular mycorrhizal fungi increased after EM addition. The relationship between microbial communities and environmental factors has been changed. The restore effect of EM increased with the increase of addition time. These results indicated that EM addition could be a good practice to restore the health of the degraded alpine grassland ecosystem.

## 1 Introduction

The grassland ecosystems play a crucial role in the world’s terrestrial ecosystems (Lyu et al., 2022). Grasslands in the Qinghai-Tibet Plateau, duo to their distinctive features, are serving as the “sensor” and “sensitive area” for climate changes in Asia and even the Northern Hemisphere (Li et al., 2015, 2019; Ren et al., 2016). Among diverse grassland types, the role of alpine grasslands figures prominently in contributing ecological services and functions (Yang et al., 2021). However, amidst the global environmental changes, long-term inappropriate human activities have led to extensive degradation of alpine grasslands (Peng et al., 2019). Studies in degraded alpine grasslands have indicated that the decomposition rate of soil organic matter was sluggish and supply of available nutrients (nitrogen, phosphorus) was deficiency, leading to sparse vegetation, reduced species diversity, compromised forage quality, and diminished grass yield (Du et al., 2020; Liu et al., 2022; Wang et al., 2022). Therefore, the effective restoration of degraded alpine grasslands was paramount not only for the preserving of the ecological environment but also for fostering sustainable regional economic development.

Previous studies have found that soil microorganisms are the most indispensable and dynamic components of the soil system (Brangarí et al., 2022; Coban et al., 2022). Soil microorganisms have significantly influenced on ecosystem processes and functions, such as nutrient cycling, plant productivity, and biodiversity (Zhang et al., 2016; Pan et al., 2023). Furthermore, soil microorganisms exhibited a heightened sensitivity to environmental changes, enabling them to respond swiftly to fluctuations of soil ecosystems and the surrounding environment, which make them be important index to evaluate soil quality and health (Coban et al., 2022). With grassland degradation progressing, the abundance of bacteria, fungi, and actinomycetes in the soil are gradually declining (Che et al., 2019; Wang et al., 2021). The degradation of grassland soil also disrupts the microbial community structure, ultimately leading to a decline in the population of beneficial soil microorganisms (Luo et al., 2022; Liao et al., 2023). As a chain reaction of microbial changes, soil fertility declines, plant productivity reduces, and ultimately a vicious cycle of degradation occurs (Che et al., 2020; Wang et al., 2021). Hence, improving the community composition and activity of soil microorganisms in degraded alpine grasslands might be an effective strategy for restoring the degrades ecosystems.

Microbial inoculants, which a new type of agricultural fertilizer developed in recent years, are formulated with one or multiple types of microorganisms (Szymanek et al., 2020). Compared to single-strain microbial inoculants, EM (Effective Microorganisms) is a distinct type of microbial inoculant that is generated through natural fermentation processes. EM consists of a diverse range of microorganisms, such as photosynthetic bacteria, lactic acid bacteria, yeast, and others. Notably, EM is recognized for its environmentally friendly characteristics (Abd El-Mageed et al., 2020). When EM is added to soil, EM has the ability to multiply microbial production, leading to the formation of dominant microbial populations (Abd El-Mageed et al., 2020). This action, in turn, enhances the activity of soil microorganisms and enzymes, promotes the decomposition of organic matter and the availability of soil nutrient (Liang et al., 2021). In addition, beneficial microorganisms in EM can produce sugars that foster the formation of soil aggregates, and reduce nutrient loss (Szymanek et al., 2020).

Currently, EM has been extensively applied and has shown promising results in tackling the problem of reduced microbial activity in degraded soil of agricultural ecosystems (Daiss et al., 2007; Megali et al., 2015). Compare to agricultural ecosystems with intensive management practices and a limited diversity of plant species, grasslands exhibited higher plant species diversity. As for alpine grasslands, their environmental conditions are harsh with intense ultraviolet radiation, low temperatures, and frequent freeze-thaw cycles (Dong et al., 2019; Li et al., 2019). Consequently, the alterations in soil physicochemical properties and microbial community structure of degraded alpine grasslands under the impact of EM might deviate from those in agricultural systems. However, the available research information remained insufficient, hence, the present study aimed to explore the impacts of varying gradients of EM on the aboveground vegetation, as well as the soil physicochemical properties and microbiota in degraded alpine grasslands. We hypothesized the following findings: (1) the addition of EM significantly increased the aboveground biomass of degraded alpine grassland, (2) the addition of EM significantly affected soil physicochemical and microbiological properties in degraded alpine grassland, and (3) The influence of EM on aboveground biomass, soil physicochemical and microbiological properties increased with the increase of EM retention time in degraded alpine grassland.

## 2 Materials and methods

### 2.1 Study site and experimental materials

The moderate-severe degraded alpine grassland selected at 2017 according to the national standard (GB19377-2003) was located in the Senduo town, the northeast part of the Qinghai-Tibetan Plateau (36°35′N, 101°42′E, 3220 m a.s.l). The climate of experimental region is typical plateau continental climate with mean annual temperature of 2.3°C and mean annual precipitation of 403.80 mm. The annual evaporation is 1378.5 mm (Li et al., 2021). The main plants in the experimental site include *Poa crymophila*, *Cleistogenes squarrosa*, *Carex tristachya*, *Elymus nutans*. *Griseb, Ligularia Cass*, *Stipa krylovii Roshev*. *Oxytropis DC*, and *Stellera chamaejasme*. L. (Li et al., 2021).

Effective microorganisms (EM) mainly include lactic acid bacteria, photosynthetic bacteria, yeast and other bacteria, which were purchased from Beijing Baifeng world Biological Technology Co. Ltd. (China). The number of contained effective viable bacteria is ≥ 10 × 10^8^ cfu⋅mL^–1^.

### 2.2 Experimental design and sampling

Effective microorganisms was diluted in a ratio of 1:4 (EM: water, v/v) before addition. The diluted EM was applied at four levels: control without EM (250 mL m^–2^ of water, CK), 150 mL m^–2^ diluted EM (M1), 200 mL m^–2^ diluted EM (M2) and 250 mL m^–2^ diluted EM (M3). The experimental design was a complete random block design with three blocks. Each experimental plot was 2 m × 2 m with 1.5 m buffer strip between each plot, and 3.0 m buffer strip was established between each blocks. In order to facilitate the colonization of EM, the combination of hole application and surface application were used to accelerate the migration of microorganisms (Li et al., 2020). In short, each plot drilled 16 holes with 3.5 cm diameter 20 cm depth, and 50 cm interval among each other. Firstly, half of the EM diluent according to treatments was added into each hole, then the rest was evenly sprayed on the surface of plot with watering can. During the experiment, the experimental station was fenced. The EM were continuously added for three years, on May 2017, 2018, and 2019, respectively.

On August, 2017, 2018, and 2019, plant and soil samples were collected. For plant samples, three quadrates with 50 cm × 50 cm were randomly selected in each plot to collect aboveground plants. The collected plant samples were oven-dry at 65°C to a constant weight and weighted. After plant collection, soil samples in the plant quadrates were collected from 0–10 to 10–20 cm soil layers, respectively, with soil auger 3.5 cm diameter. The same layer of soil sample in each plot was composted and then separated into three parts: one part was used to test soil water content (SWC), one part was dried at 65°C for soil physiochemical analysis, and the third part was stored at −20°C for soil microorganism analysis.

### 2.3 Soil samples measurement

The soil samples for measuring SWC were put into aluminum box and dried at 65°C to constant weight to determine soil water content (SWC). Soil samples for measuring soil physicochemical properties were determined soil total nitrogen (TN), soil organic carbon (TOC), soil pH, soil electric conductivity (EC), NH_4_^+^-N, NO_3_^–^-N, and soil available phosphorus (AP). TN and TOC were measured by Fisher 2000 elemental analyzer (Thermo Fisher Scientific, Italy). Soil pH and EC were measured using the acidity meter and conductivity meter (METTLER TOLEDO, Switzerland). NH_4_^+^-N and NO_3_^–^-N was determined by flow autoanalyzer (FIA Compact, Germany). Soil available phosphorus (AP) was measured by sodium bicarbonate extraction molybdenum antimony anti-colorimetric method. The microorganisms in soil were measured by phospholipid fatty acid (PLFA) which modified by White et al. (1979). Briefly, the 8 g of freeze-dried soil were added to the single-phase mixture of chloroform, methanol and phosphate buffer (1:2:0.8) and extract lipids. The crude extracts were isolated and concentrated by silicic acid column chromatography. Then, the phospholipid fatty acid methyl esters (FAMEs) were obtained by saponifying and methylating. the FAMEs was analyzed by the gas chromatography (Agilent 6850, USA). Then, PLFAs were defined using the Sherlock MIDI software (Newark, DE, USA), and PLFAs classification was shown in Supplementary Table 1.

### 2.4 Statistical analysis

The differences of plant biomass, soil physicochemical properties and microorganisms between treatments and years were confirmed by ANOVA. the principal component analysis (PCA) was used to analyze the soil microbial community profiles (PLFAs) in treatments at different years in vegan package. The relationships between microorganisms and environmental variables was analyzed using the redundant analysis (RDA) with vegan package. Pearson correlation was used to determine the correlation coefficients between soil physical and chemical properties. The relationship between microbial communities (PLFAs) and soil factors of different treatments was compared by mantel test. The PCA (Bray-Curtis) results was used to perform Procrustes analysis. All statistical analyses were calculated in R 4.0.2. The significance for all statistical tests was *P* < 0.05.

## 3 Results

### 3.1 Changes of aboveground vegetation biomass under EM addition

**FIGURE 1:**
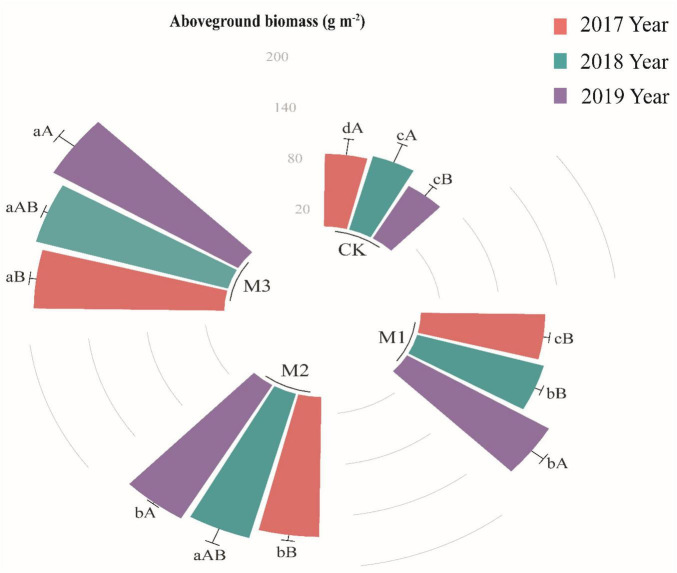
The variations of aboveground biomass (g m^– 1^) at different treatments in 2017, 2018, and 2019. Values are mean ± standard error. Values followed by a different lowercase letter are significant difference among treatments, values followed by a different capital letter are significant difference between years at *P* < 0.05.

Compared with CK, the aboveground biomass in the degraded alpine grassland significantly increased with the addition of EM, (Figure 1), moreover, EM adding amount and time had positive effect on the aboveground biomass (Figure 1). It indicated that EM addition had cumulative effect to some extent. The maximum aboveground biomass was in M3 of different years, which were 193.68, 202.71, and 218.44 g m^–2^ in 2017, 2018, and 2019, respectively (Figure 1).

### 3.2 Changes of soil physicochemical properties under EM addition

Compared to CK, TOC and TN significantly increased after the addition of EM, particularly in the high addition of EM (M2, M3). In the 10–20 cm soil layer, the effect of EM addition on TOC and TN decreased, resulting in a relatively smaller increase (Table 1). Across different years, TOC or TN had no significant differences in the same EM addition (Table 1). Compared to NO_3_^–^-N, the addition of EM had a more pronounced effect on increasing NH_4_^+^-N. Interestingly, across different years, the M2 consistently showed the highest NH_4_^+^-N, 26.75, 50.41, and 28.77 mg kg^–1^ in 2017, 2018, and 2019, respectively (Table 1). In the same year, NO_3_^–^-N had no significant changes among different treatments. However, with increasing experimental time, NH_4_^+^-N showed an initial increase then followed a decrease, whereas NO_3_^–^-N exhibited a decline initially and then an increase (Table 1). The addition of EM significantly influenced AP, especially in the 0–10 cm soil layer. Similar to the effect on NH_4_^+^-N, the M2 consistently exhibited significantly higher of AP compared to the other treatments, 3.70, 3.39, 3.16 mg kg^–1^ in 2017, 2018, and 2019, respectively (Table 1). AP within the same treatment showed a declining trend as the experimental time increased.

**TABLE 1:** Variations of TOC (soil total organic C), TN (total N), NH_4_^+^-N, NO_3_^–^-N, and AP (available P) at the two soil layers under different EM input in 2017, 2018, and 2019.

Parameter	Year	Layer (cm)	CK	M1	M2	M3
TOC	2017	0–10	33.14 ± 1.63bB	34.58 ± 5.03bA	43.86 ± 5.42aA	39.56 ± 4.49aA
(g kg^–1^)	2018		40.92 ± 1.62abA	38.79 ± 1.68bA	46.04 ± 3.75aA	40.44 ± 1.05bA
	2019		34.26 ± 1.04bB	35.08 ± 3.36bA	43.37 ± 1.74aA	43.73 ± 0.87aA
	2017	10–20	30.16 ± 1.4aA	29.74 ± 2.27aA	29.29 ± 2.63aA	34.17 ± 1.91aB
	2018		30.45 ± 0.68aA	29.96 ± 1.26aA	32.78 ± 3.61aA	32.95 ± 0.55aB
	2019		30.19 ± 0.21bA	34.78 ± 2.3aA	28.91 ± 1.58bA	37.49 ± 1.10aA
TN	2017	0–10	2.76 ± 0.12bB	3.18 ± 0.23abAB	3.69 ± 0.21aA	3.67 ± 0.27aA
(g kg^–1^)	2018		3.43 ± 0.26bA	3.49 ± 0.24abA	4.00 ± 0.25aA	3.64 ± 0.02abA
	2019		3.17 ± 0.24bAB	2.90 ± 0.20bB	3.74 ± 0.07aA	3.84 ± 0.30aA
	2017	10–20	2.40 ± 0.18aB	2.58 ± 0.17aA	2.63 ± 0.37aA	2.78 ± 0.19aA
	2018		3.03 ± 0.20aA	2.83 ± 0.21aA	3.19 ± 0.47aA	3.51 ± 0.71aA
	2019		2.69 ± 0.2aAB	2.78 ± 0.70aA	2.65 ± 0.22aA	3.33 ± 0.27aA
NH_4_^+^-N	2017	0–10	18.33 ± 0.83cC	21.95 ± 2.96bB	26.75 ± 1.58aB	23.13 ± 2.22bB
(mg kg^–1^)	2018		35.05 ± 3.67bcA	31.61 ± 5.98cA	50.41 ± 5.56aA	43.15 ± 1.73abA
	2019		18.13 ± 1.20cB	21.10 ± 2.47bB	28.77 ± 3.31aB	24.39 ± 2.31abB
	2017	10–20	16.71 ± 1.50bB	24.95 ± 2.18aA	25.97 ± 2.44aA	23.96 ± 1.31aA
	2018		22.17 ± 1.31aA	15.88 ± 3.79bB	12.33 ± 2.39bB	16.42 ± 2.04abB
	2019		14.64 ± 2.11aB	11.59 ± 1.31abB	10.26 ± 0.62bB	9.27 ± 0.93bC
NO_3_^–^-N	2017	0–10	7.57 ± 1.56aAB	7.41 ± 0.96aB	6.99 ± 1.18aAB	6.47 ± 0.47aB
(mg kg^–1^)	2018		4.97 ± 0.70bB	5.58 ± 2.17bB	4.91 ± 0.92bB	7.51 ± 0.33aB
	2019		8.96 ± 1.72bA	11.69 ± 0.25bA	9.29 ± 0.74bA	18.25 ± 4.49aA
	2017	10–20	7.18 ± 1.08aAB	6.31 ± 0.32aB	8.00 ± 1.07aA	7.95 ± 0.79aA
	2018		4.94 ± 0.79bB	8.66 ± 0.76aA	8.73 ± 0.51aA	9.09 ± 0.56aA
	2019		11.09 ± 2.99aA	10.12 ± 0.64aA	9.62 ± 0.15aA	9.90 ± 2.19aA
AP	2017	0–10	2.68 ± 0.24bA	3.66 ± 0.23aA	3.70 ± 0.22aA	2.91 ± 0.36bB
(mg kg^–1^)	2018		3.02 ± 0.24aA	2.93 ± 1.38bB	3.39 ± 1.14aB	3.27 ± 0.95abA
	2019		1.57 ± 0.17bB	2.53 ± 0.48aB	3.16 ± 0.20aB	2.79 ± 0.10aB
	2017	10–20	1.79 ± 0.13bA	2.47 ± 0.46abA	2.98 ± 0.98aA	2.83 ± 0.24abAB
	2018		1.73 ± 0.36bA	1.80 ± 0.49bA	2.97 ± 0.42bB	2.08 ± 0.35aA
	2019		1.49 ± 0.03bA	2.28 ± 0.23aA	2.45 ± 0.26aAB	2.24 ± 0.19aB

Values are mean ± standard error. Lowercase letters indicate significant difference among treatments, and uppercase letters indicate significant difference between years at *P* < 0.05. CK means control without EM, M1 means the addition of 150 mL m^–2^ diluted EM, M2 means the addition of 200 mL m^–2^ diluted EM and M3 means the addition of 250 mL m^–2^ diluted EM.

The addition of EM exhibited a delayed impact on pH. There were no notable changes in pH across the different treatments in 2017 and 2018. However, the addition of EM significantly increased the pH in 2019. As the experimental time increased, a gradual increase in pH occurred across the different treatments (Table 2). The addition of EM had a more rapid and pronounced effect on EC compared to pH. EC had a significant reduction in the 10–20 cm soil layers. Furthermore, EC showed no significant changes across different EM addition treatments at the same years (Table 2). In the 0–10 cm soil layer, the high addition of EM (M3) significantly improved SWC. Furthermore, the addition of EM resulted in a reduction in the C/N compared to CK, although these changes were not statistically significant (Table 2).

**TABLE 2:** Variations of pH, EC (electrical conductivity), SWC (soil water content), and C/N (soil total organic C: total N) at the two soil layers under different EM input in 2017, 2018, and 2019.

Parameter	Year	Layer (cm)	CK	M1	M2	M3
pH	2017	0–10	7.12 ± 0.05aB	7.20 ± 0.08aC	7.09 ± 0.13aC	6.99 ± 0.08aB
	2018		7.55 ± 0.03aA	7.59 ± 0.18aB	7.53 ± 0.14aB	7.63 ± 0.09aA
	2019		7.28 ± 0.22bAB	7.97 ± 0.06aA	7.95 ± 0.07aA	7.77 ± 0.13aA
	2017	10–20	7.24 ± 0.11aB	7.25 ± 0.21aB	7.26 ± 0.09aC	7.28 ± 0.12aB
	2018		7.48 ± 0.09aA	7.70 ± 0.16aA	7.64 ± 0.09aB	7.65 ± 0.07aA
	2019		7.49 ± 0.05cA	7.97 ± 0.03aA	8.02 ± 0.13aA	7.78 ± 0.01bA
EC	2017	0–10	362.76 ± 46.31aB	151.4 ± 42.31bA	179.67 ± 31.27bA	163.87 ± 27.02bA
	2018		574.09 ± 28.54aA	194.57 ± 17.4bA	205.43 ± 13.63bA	186.53 ± 16.66bA
	2019		209.80 ± 21.2aC	196.20 ± 17.31aA	191.1 ± 19.61aA	208.83 ± 32.09aA
	2017	10–20	324.91 ± 11.21aB	175.73 ± 34.68bA	183.5 ± 36.58bA	161.80 ± 28.45bA
	2018		605.27 ± 99.51aA	191.97 ± 15.37bA	199.47 ± 14.20bA	202.87 ± 26.05bA
	2019		197.53 ± 14.41aB	162.87 ± 11.46aA	173.4 ± 16.08aA	171.83 ± 25.92aA
SWC (%)	2017	0–10	24.52 ± 0.93aA	24.29 ± 2.52aA	22.78 ± 2.05aA	26.97 ± 1.9aA
	2018		18.82 ± 0.38bB	15.44 ± 0.88bB	18.86 ± 1.15bB	24.86 ± 2.41aA
	2019		16.46 ± 1.01bC	19.52 ± 2.06bB	18.50 ± 1.42bB	24.18 ± 0.77aA
	2017	10–20	21.14 ± 0.71bA	20.73 ± 1.68bA	20.94 ± 2.06bA	25.71 ± 1.87aA
	2018		19.85 ± 0.51abA	17.72 ± 0.68bAB	19.68 ± 1.62abA	21.43 ± 1.65aB
	2019		16.08 ± 0.81aB	17.17 ± 1.33aB	17.26 ± 0.83aA	16.86 ± 0.94aC
C/N	2017	0–10	12.02 ± 0.49aA	10.83 ± 0.85aA	11.85 ± 0.79aA	10.85 ± 1.42aA
	2018		12.02 ± 1.38aA	11.15 ± 0.74aA	11.51 ± 0.53aA	11.11 ± 0.31aA
	2019		12.55 ± 0.57aA	12.02 ± 0.59aA	12.27 ± 0.97aA	11.08 ± 0.77aA
	2017	10–20	12.68 ± 1.38aA	11.53 ± 0.15aA	11.20 ± 0.59aA	12.33 ± 0.68aA
	2018		10.08 ± 0.47aB	10.60 ± 0.38aA	10.32 ± 0.37aA	9.70 ± 1.59aA
	2019		11.28 ± 0.80aAB	13.39 ± 3.51aA	10.95 ± 0.69aA	11.34 ± 0.92aA

Values are mean ± standard error. Lowercase letters indicate significant difference among treatments, and uppercase letters indicate significant difference between years at *P* < 0.05. CK means control without EM, M1 means the addition of 150 mL m^–2^ diluted EM, M2 means the addition of 200 mL m^–2^ diluted EM and M3 means the addition of 250 mL m^–2^ diluted EM.

### 3.3 Changes of soil microorganisms under EM addition

**FIGURE 2:**
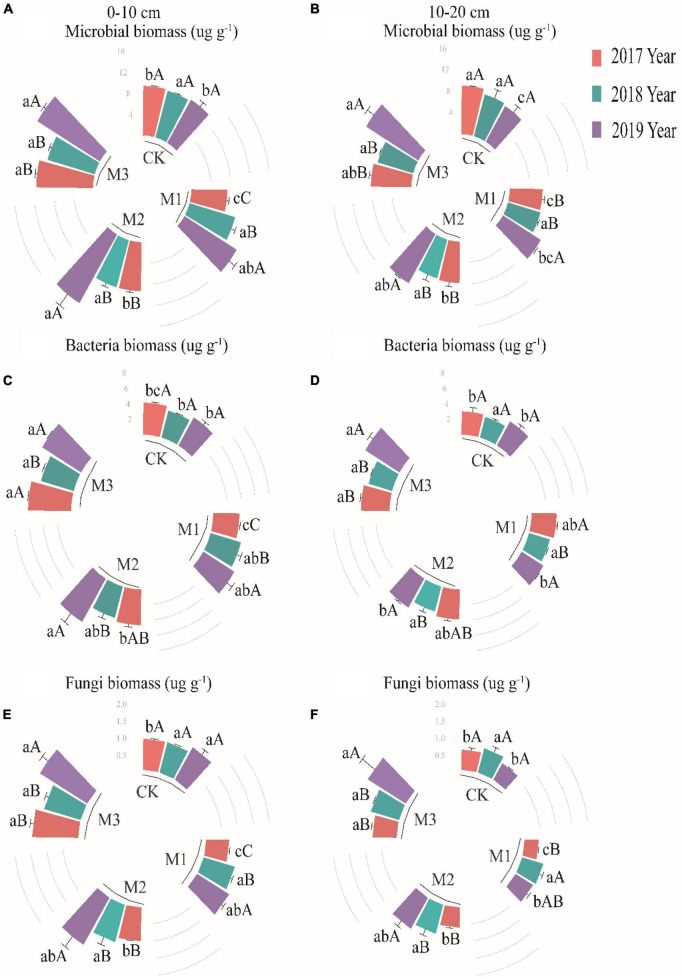
The variations of microbial biomass **(A,B)**, bacteria **(C,D)** and fungi **(E,F)** at two soil layers under different treatments in 2017, 2018, and 2019. Values are mean ± standard error. Values followed by a different lowercase letter are significant difference among treatments, values followed by a different capital letter are significant difference between years at *P* < 0.05.

The linear relationship between EM gradients and microbial biomass variables was weak (Supplementary Figure 1). Total microbial biomass in the 0–10 cm soil layer showed relatively small variations among different treatments in 2017 and 2018, but significantly enhanced in 2019. The highest microbial biomass was observed in the M3, which was 58.84% higher compared to CK (Figure 2A). In 2019, the microbial biomass significantly increased with the increase of EM addition in the 10–20 cm soil layer, increasing from 8.91 ng g^–1^ (M1) to 10.63 ng g^–1^ (M2) to 12.28 ng g^–1^ (M3) (Figure 2B). The addition of EM significantly increased the bacteria biomass in the 0–10 cm soil layer. Compared to CK, the bacteria biomass increased by 46.97% (2017), 32.31% (2018), and 54.52% (2019), respectively. Additionally, the bacteria biomass in the EM treatments showed a significant increase as the time increased (Figure 2C). In the 10–20 cm soil layer, the bacteria biomass showed a significant increase only in M3 treatment of 2019 as compared to other treatments (Figure 2D). With the addition of EM, the fungi biomass showed a significant increase with the increase of EM (except in 2018), and the maximum values appeared in the M3, with values of 1.43 ng g^–1^ (2017), 1.18 ng g^–1^ (2018), and 1.71 ng g^–1^ (2019), respectively (Figure 2E). In the 10–20 cm soil layer, the fungi biomass in the high EM addition treatment (M3) were significantly higher than in other treatments (Figure 2F).

**FIGURE 3:**
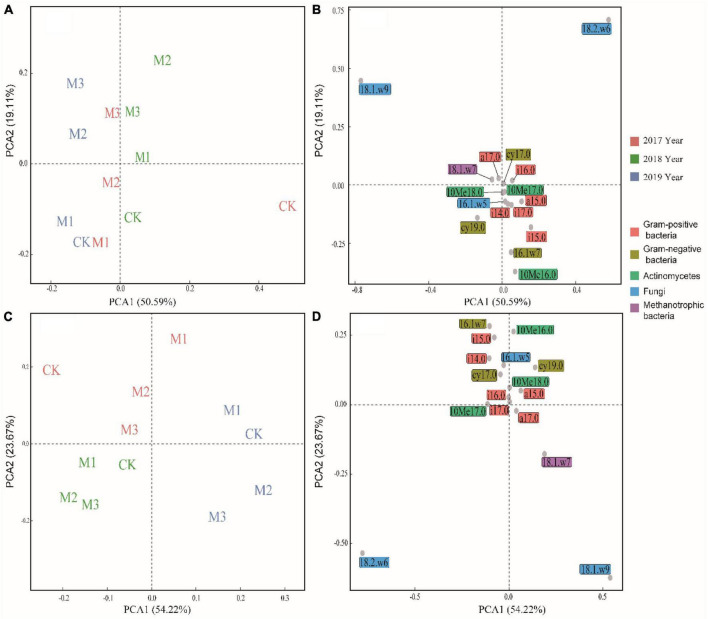
Principal component analysis (PCA) of microbial community at two soil layers. **(A,B)**: 0–10 cm soil layer, **(C,D)**: 10–20 cm soil layer.

Through PCA analysis, in the 0–10 cm soil layer, the relative contents of 18.1.w9c, 18.2.w6, 9c, a17.0, i16.0, and 18.1.w7c were higher in the treatments with higher EM addition (M2, M3) than those in the CK, whereas the relative contents of i15.0, 16.1w7c, and 10Me16.0 were higher in the CK than those in M2 and M3 (Figures 3A, B). In the 10–20 cm soil layer, the impact of experimental time on microbial community structure outweighed the influence of EM addition. In 2019, there was a noticeable increase in the relative content of 18.1.w9c, 18.2.w6, 9c, and 18.1.w7c, particularly in M2 and M3 (Figures 3C, D). The addition of EM significantly increased the F:B, especially in M3. Moreover, with the increase of experimental time, the F:B showed an upward trend. In contrast to F:B, the addition of EM significantly decreased GP:GN, particularly in the 0–10 cm soil layer (Table 3). In the 0–10 cm soil layer, the addition of EM significantly increased MB:TOC and MB:TN, especially in M3. And with the increase of time, MB:TOC and MB:TN increased (Table 3).

**TABLE 3:** Variations in F:B (Fungi:bacteria), GP:GN (Gram-positive:Gram-negative), MB:TOC (microbial biomass:total organic carbon), and MB:TN (microbial biomass:total nitrogen) at the two soil layers under different EM input in 2017, 2018, and 2019.

Parameter	Year	Layer (cm)	CK	M1	M2	M3
F:B	2017	0–10	0.22 ± 0.02abB	0.20 ± 0.01bA	0.22 ± 0.02abB	0.24 ± 0.01aA
	2018		0.22 ± 0.01bA	0.23 ± 0.02aA	0.27 ± 0.02aA	0.25 ± 0.03aA
	2019		0.20 ± 0.01bB	0.24 ± 0.03abA	0.24 ± 0.01abA	0.27 ± 0.04aA
	2017	10–20	0.20 ± 0.01aAB	0.13 ± 0.01bB	0.15 ± 0.01bB	0.19 ± 0.02aA
	2018		0.26 ± 0.04aA	0.22 ± 0.03aA	0.26 ± 0.01aA	0.27 ± 0.01aA
	2019		0.16 ± 0.02bB	0.18 ± 0.02aA	0.24 ± 0.05aA	0.24 ± 0.07aA
GP:GN	2017	0–10	0.74 ± 0.03aB	0.62 ± 0.01bB	0.65 ± 0.07abA	0.61 ± 0.02bB
	2018		0.80 ± 0.02aB	0.61 ± 0.04bB	0.68 ± 0.04bA	0.64 ± 0.02bB
	2019		1.28 ± 0.06aA	0.78 ± 0.01bA	0.76 ± 0.04bA	0.70 ± 0.01bA
	2017	10–20	0.73 ± 0.11aB	0.88 ± 0.09aA	0.77 ± 0.06aA	0.75 ± 0.1aA
	2018		0.74 ± 0.02bB	0.75 ± 0.01bA	0.84 ± 0.02aA	0.83 ± 0.03aA
	2019		1.11 ± 0.08aA	0.80 ± 0.05bA	0.82 ± 0.08bA	0.78 ± 0.08bA
MB:TOC	2017	0–10	0.28 ± 0.01aA	0.21 ± 0.02bB	0.21 ± 0.03bB	0.30 ± 0.04aAB
	2018		0.22 ± 0.01abB	0.25 ± 0.03aB	0.20 ± 0.01bB	0.25 ± 0.02aB
	2019		0.27 ± 0.02bA	0.35 ± 0.05aA	0.34 ± 0.05aA	0.34 ± 0.01aA
	2017	10–20	0.30 ± 0.01aA	0.22 ± 0.01bB	0.26 ± 0.03abB	0.24 ± 0.01bB
	2018		0.26 ± 0.05aA	0.23 ± 0.01aB	0.23 ± 0.03aB	0.23 ± 0.01aB
	2019		0.25 ± 0.04bA	0.26 ± 0.01bA	0.37 ± 0.02aA	0.33 ± 0.05aA
MB:TN	2017	0–10	3.36 ± 0.11aA	2.30 ± 0.12bB	2.49 ± 0.22bB	3.16 ± 0.34abB
	2018		2.65 ± 0.19aB	2.75 ± 0.14aB	2.28 ± 0.07bB	2.73 ± 0.10aB
	2019		2.96 ± 0.08bA	4.28 ± 0.71aA	3.98 ± 0.65ab	3.85 ± 0.29abA
	2017	10–20	3.78 ± 0.33aA	2.58 ± 0.09bA	2.95 ± 0.39bB	2.95 ± 0.11bAB
	2018		2.67 ± 0.56aB	2.42 ± 0.10aA	2.36 ± 0.31aB	2.21 ± 0.43aB
	2019		2.84 ± 0.31bAB	3.41 ± 0.81abA	4.04 ± 0.37aA	3.74 ± 0.71aA

Values are mean ± standard error. Lowercase letters indicate significant difference among treatments, and uppercase letters indicate significant difference between years at *P* < 0.05. CK means control without EM, M1 means the addition of 150 mL m^–2^ diluted EM, M2 means the addition of 200 mL m^–2^ diluted EM and M3 means the addition of 250 mL m^–2^ diluted EM.

### 3.4 The interaction between microorganisms and soil properties

**FIGURE 4:**
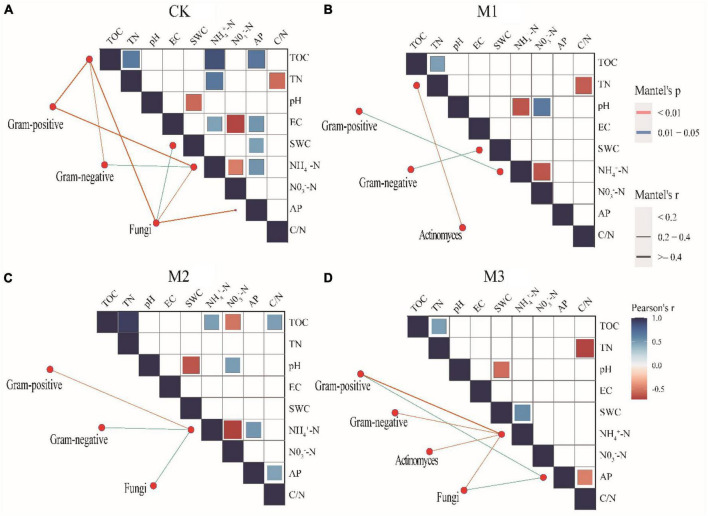
Pairwise comparisons between environmental factors and microbial communities by partial Mantel tests for different treatments. The color gradients denote Spearman’s correlation coefficients; line edge width corresponds to the Mantel’s r statistic for the corresponding distance correlations, and edge color denotes the statistical significance based on 9,999 permutations. **(A)** 250 mL m-2 of water; CK; **(B)** 150 mL m-2 diluted EM (M1); **(C)** 200 mL m-2 diluted EM (M2); **(D)** 250 mL m-2 diluted EM (M3).

**FIGURE 5:**
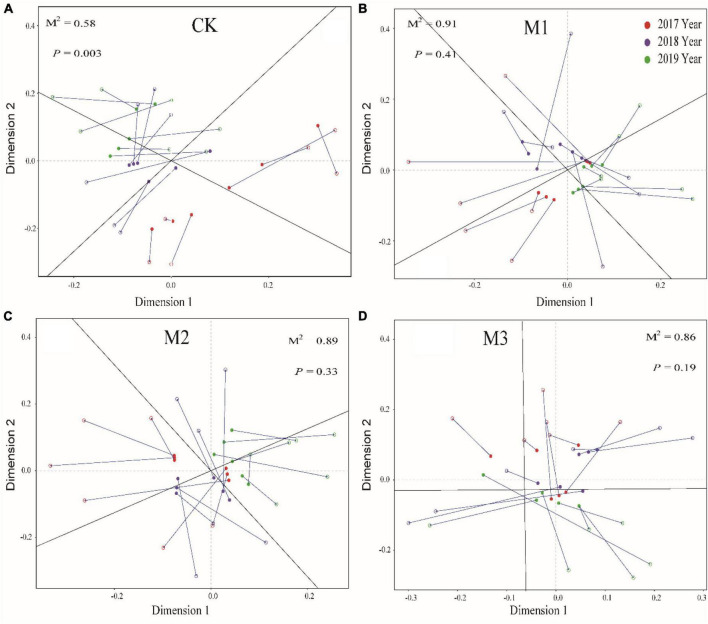
Procrustes analysis of the correlation between soil physicochemical properties and microbial community based on the PCA (Bray-Curtis) results of different treatments. **(A)** 250 mL m-2 of water; CK; **(B)** 150 mL m-2 diluted EM (M1); **(C)** 200 mL m-2 diluted EM (M2); **(D)** 250 mL m-2 diluted EM (M3).

The addition of EM reduced the correlation of soil factors, especially in M3 (Figure 4). Moreover, Mental test found that compared to CK, the addition of EM also reduced the correlation between different microbial communities and soil factors (Figure 4). The result was further supported by Procrustes analysis, which revealed that the addition of EM resulted in *P* > 0.05. This suggested that the coherence between soil characteristics and microbial communities in treatments with EM addition (M1, M2, and M3) was less pronounced compared to the CK (Figure 5).

**FIGURE 6:**
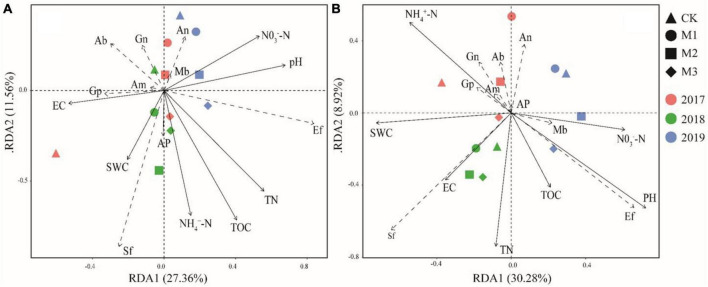
Redundancy analysis (RDA) of environmental variables and indicator PLFAs. **(A)**: 0–10 cm soil layer, **(B)**: 10–20 cm soil layer. black lines represented environmental variables, and dashed line represented indicator PLFAs. See Tables 1, 2 and Supplementary Table 1 for abbreviations.

In RDA, the influence of EC, SWC and AP on the microbial community structure was smaller than other soil factors in the 0–10 cm soil layer. SF and EF had a positive correlation with most soil factors (Figure 6A). The addition of EM increases the sensitivity of microorganisms to soil factors in 10–20 cm soil layer, and the influence of TN, NH_4_^+^-N, and pH on the microbial community structure was higher than other soil factors (Figure 6B).

## 4 Discussion

Effective microorganisms application caused positive effects on the ecosystem of degraded alpine grassland. with increasing the application amount and experimental time of EM, the aboveground biomass of degraded alpine grassland significantly increased (Figure 1). The addition of EM led to the establishment of beneficial microbial populations in the soil which not only improved its water-holding and nutrient-retention and available capacities but also stimulated the activity of microorganisms and soil enzymes (Table 1 and Figure 2). These positive changes accelerate the decomposition of organic matter, and make it easier for plants to uptake nutrients (Jin et al., 2022; Xiao et al., 2022). In addition, the beneficial microorganisms in EM have the ability of inhibiting harmful pathogens, and can also decompose cellulose and lignin and transform them into available nutrients for plants (Liu et al., 2021; Khan, 2022). Furthermore, beneficial microorganisms can decompose some toxic substances such as sulfides and make plants to grow in a favorable environment (Elnahal et al., 2022; Nikolaou et al., 2023).

Our study revealed a significant increase of TOC, TN, and AP with any addition of EM in the 0–10 cm soil layer of degraded alpine grassland. Nevertheless, in the 10–20 cm soil layer, a significant increase was only observed in M3 (Table 1). This indicated that a higher addition of EM was more conducive to the accumulation of TOC, TN, and AP. Compared with agricultural land, the degraded alpine grassland has unique ecological environments and the sod layer formed by interweaving live roots of different ages and dead roots (Liu et al., 2023). The sod layer contains a large amount of organic matter, and the use of microbe agents can increase the number and activity of microorganisms in the soil (Jin et al., 2022), and then enhanced the decomposition of organic matter in the sod layer, leading to an increase in TOC, TN, and AP. However, in deeper soil layers, the addition of EM through hole application may not have evenly distributed throughout soil layers due to soil structure. This may lead to a greater soil nutrient increase in surface soil compared to deep soil. Furthermore, alterations in the composition of soil microorganisms can significantly influence the rate of decomposition of organic matter. Some studies have demonstrated that an increased F:B and a decreased GP:GN have a positive effect on the decomposition of soil organic matter (Kumar and Ghoshal, 2017; Fanin et al., 2019), which align with the findings of this study (Table 3). Interestingly, our research revealed that the M2, rather than the M3, had the maximum TOC, TN, and AP (Table 1). In M1, the reduced capacity for organic matter decomposition could potentially be attributed to a lower addition of EM, leading to lower TOC, TN, and AP relative to those in M2 and M3. Microorganisms and plants require a large quantity of nutrients to be absorbed from soil in order to maintain their own growth and development (Cheeke et al., 2021; Rosinger et al., 2022), and the high activity levels of microorganisms escalate the depletion of soil carbon substrates (Chen et al., 2003). This study found that the aboveground biomass, microbial biomass, MB:TOC and MB:TN in M3 were all maximum (Figures 1, 2 and Table 3). Thus, although the highest addition of EM in M3, it might also consume more nutrients, resulting in not the highest of TOC, TN, and AP.

The addition of EM increased available nitrogen in the soil, and the content of NH_4_^+^-N was higher than NO_3_^–^-N (Table 1). Plants tend to absorb NO_3_^–^-N from the soil for their growth, then resulting in a reduction of the net residual amount of nitrate nitrogen in the soil (Kant, 2018). Moreover, negatively charged NO_3_^–^-N was more susceptible to leaching than positively charged NH_4_^+^-N. Consequently, the accumulation of NH_4_^+^-N in the soil is generally higher compared to NO_3_^–^-N (Latifah et al., 2017). However, NH_4_^+^-N or NO_3_^–^-N generally increased after the addition of EM. This indicated that the EM alleviated the limitation of available nitrogen in the degraded alpine grassland to some extent, and was beneficial for the growth of vegetation and microorganisms. Soil pH increased with the addition of EM and the increase of experimental time (Table 2). This could potentially be linked to a range of chemical reactions facilitated by EM addition. Specifically, the decarboxylation process of releasing OH^–^ during organic carbon mineralization and the proton action of digesting H^+^ all increase pH (Laurent et al., 2020). Slight increase of pH is beneficial for reducing the effectiveness of copper and zinc ions, which is more favorable for plant growth and soil microbial activity (Laurent et al., 2020). The increase in the dosage and time of EM significantly reduced the EC (Table 2). EM can boost the activity of beneficial microorganisms, leading to the production of polysaccharides and adhesives that form soil binders. This, in turn, can impact the structure of aggregates, leading to a decrease in bulk density and non-capillary pores. As a result, soil leaching is hastened while soil salinity is correspondingly reduced (Bello et al., 2021; Chen et al., 2022).

The high addition of EM, such as M2 and M3, resulted in a significantly higher amount of soil microorganisms, bacteria, and fungi compared to the CK, particularly in 2019 (Figure 2). The addition of EM might result in competition with those indigenous microorganisms and hinder the growth and development of specific pathogens (Mallon et al., 2015; Shi et al., 2022). All of these activity would cause alterations in the composition of microorganisms in the soil. On the other hand, it is imperative to recognize the presence of a colonization issue after the addition of EM (Niu et al., 2020), and the hostile soil environment in alpine grasslands might hinder the survival of introduced microbes, ultimately impacting their efficacy levels and outcomes. A study found that the environmental alterations stemming from microorganisms in inoculants necessitated a specific duration for their manifestation (Griffiths and Philippot, 2013). Similarly, changes in microbial diversity also demand a particular temporal sequence for their occurrence. Therefore, in the first 2 years of the study (2017, 2018), The microorganisms in EM might undergo significant substitution, transformation, and adaptation processes as they interact with the indigenous microorganisms. By Procrustes analysis and regression analysis, it revealed that the addition of EM resulted in an incongruity between the microbial community and the soil environment (Figure 5), and there was no statistically significant correlation between the dosage of EM addition and the alterations in microbial biomass (Supplementary Figure 1). In the third year of the study, the microbial population in EM and the indigenous microorganisms might exhibit a marked adaptation to the altered microenvironment, ultimately facilitating the attainment of a state of ecosystemic stability manifesting through the development of a resilient and enduring microbial community. The addition of EM also increased the fungal indicators (18.1.w9, 18.2.w6) and reduced gram-negative bacterial indicators (cy19.0, 16.1w7). This indicated that the microbial community experienced fluctuating changes in response to EM addition across different years. Surprisingly, despite not being intentionally introduced fungi in EM, the analysis revealed a significant increase of the fungi in degraded alpine grassland due to the influence of EM. This could be because the addition of EM enhanced the nutrient richness of the soil and mitigated any inadequacies in nitrogen, and phosphorus. These mechanisms cooperated to stimulate the growth and progression of fungi. In turn, the higher 18.1.w9 and 18.2.w6 have been found to facilitate the breakdown of compounds like cellulose, lignin, hemicellulose, and chitin (Yu et al., 2009; Joergensen, 2021). This was conducive to the rapid and effective improvement of the soil fertility in degraded alpine grassland soil. This meant that boosting the dosage of EM could have a positive impact on the growth of beneficial microorganisms in degraded alpine grasslands, ultimately facilitating the restoration of degraded alpine grasslands.

Soil carbon and nitrogen showed the greatest impact on the structure of the soil microbial community (Figure 6), suggesting that the addition of EM enhanced the influence of C, N on soil microorganisms. In degraded grasslands, the decrease in soil organic carbon and nitrogen is found to be the primary cause of reduced microbial activity (Zeng et al., 2021; Yang et al., 2023). In this study, the addition of EM increased soil carbon and nitrogen (Table 1), and the structure of soil microbial communities surely changed following the changes of carbon and nitrogen (Guo et al., 2019). Besides carbon and nitrogen, pH also significantly influenced the composition of microbial communities (Figure 6). Previous studies have also indicated that pH changes have significant impacted on different PFLAs (Rousk et al., 2009, 2010). This indicated that the addition of EM not only directly affected the changes of soil microorganisms, but also enhanced its effects on soil microorganisms through the modification of soil physicochemical parameters. Additionally, a positive correlation occurred between a majority of the environmental factors and ectomycorrhizal fungi and saprophytic fungi. This suggested that ectomycorrhizal and saprophytic fungi were particularly vulnerable to soil environments alterations in comparison to other indicator microorganisms, and they possessed a competitive edge (Li et al., 2021). The Mantel test revealed a significant correlation between NH_4_^+^-N and different microbial communities. This result verified the findings of previous studies that microorganisms prefer to use ammonium nitrogen compared to nitrate nitrogen (Mooshammer et al., 2014).

## 5 Conclusion

The addition of effective microorganisms (EM) had a significant positive effect on aboveground biomass, the soil property indexes and microorganisms in the degraded alpine grassland. The higher amount of EM addition, the more significance the improvement effect of aboveground biomass, TOC, TN, available nitrogen (NH_4_^+^-N, NO_3_^–^-N), AP and microbial biomass in the 0–20 cm soil layer, especially in 2019. EC and C/N decreased significantly after EM addition. The structure of microbial community and the responses to soil changed after EM addition. The relative biomasses of 18.1.w9c and 18.2.w6, 9c increased, and cy19.0 and 16.1w7 decreased. All effects were stronger in the 0–10 cm layer than in the 10–20 cm layer, increasing with the increase of application time. These results indicated that EM had good potential in improving aboveground biomass, soil fertility and microbial biomass of degraded alpine grassland especially in high dosage.

## Data availability statement

The datasets presented in this article are not readily available because the datasets generated during and/or analyzed during the current study are available from the corresponding author on reasonable request. Requests to access the datasets should be directed to JL, l628js@126.com.

## Author contributions

JL: Writing – original draft, Writing – review and editing. JW: Writing – review and editing. XS: Writing – original draft, Writing – review and editing. XY: Writing – original draft, Writing – review and editing. kL: Conceptualization, Funding acquisition, Writing – original draft, Writing – review and editing.

## References

[B1] Abd El-Mageed T. A. Rady M. M. Taha R. S. Abd El Azeam S. Simpson C. R. Semida W. M. (2020). Effects of integrated use of residual sulfur-enhanced biochar with effective microorganisms on soil properties, plant growth and short-term productivity of *Capsicum annuum* under salt stress. *Sci. Hortic.* 261:108930.

[B2] Bello S. K. Alayafi A. H. Al-Solaimani S. G. Abo-Elyousr K. A. M. (2021). mitigating soil salinity stress with gypsum and bio-organic amendments: A review. *Agronomy* 11:1735.

[B3] Brangarí A. G. Lyonnard B. Rousk J. (2022). Soil depth and tillage can characterize the soil microbial responses to drying-rewetting. *Soil Biol. Biochem.* 173:108806.

[B4] Che R. Liu D. Qin J. Wang F. Wang W. Xu Z. (2020). Increased litter input significantly changed the total and active microbial communities in degraded grassland soils. *J. Soils Sediments* 20 2804–2816.

[B5] Che R. Wang Y. Li K. Xu Z. Hu J. Wang F. (2019). Degraded patch formation significantly changed microbial community composition in alpine meadow soils. *Soil Tillage Res.* 195:104426.

[B6] Cheeke T. E. Schneider M. Saify A. Brauner M. Bunn R. (2021). Role of soil biota in grassland restorations in high nutrient soils. *Restor. Ecol.* 30:e13549.

[B7] Chen C. Lv Q. Tang Q. (2022). Impact of bio-organic fertilizer and reduced chemical fertilizer application on physical and hydraulic properties of cucumber continuous cropping soil. *Biomass Convers. Bioref.*

[B8] Chen G. Zhu H. Zhang Y. (2003). Soil microbial activities and carbon and nitrogen fixation. *Res. Microbiol.* 154 393–398. 12892845 10.1016/S0923-2508(03)00082-2

[B9] Coban O. De Deyn G. B. van der Ploeg M. (2022). Soil microbiota as game-changers in restoration of degraded lands. *Science* 375:abe0725. 10.1126/science.abe0725 35239372

[B10] Daiss N. Lobo M. G. Socorro A. R. Brückner U. Heller J. Gonzalez M. (2007). The effect of three organic pre-harvest treatments on Swiss chard (*Beta vulgaris* L. var. cycla L.) quality. *Eur. Food Res. Technol.* 226 345–353.

[B11] Dong S. Zhang J. Li Y. Liu S. Dong Q. Zhou H. (2019). Effect of grassland degradation on aggregate-associated soil organic carbon of alpine grassland ecosystems in Qinghai-Tibetan Plateau. *Eur. J. Soil Sci.* 71 69–79.

[B12] Du C. Jing J. Shen Y. Liu H. Gao Y. (2020). Short-term grazing exclusion improved topsoil conditions and plant characteristics in degraded alpine grasslands. *Ecol. Indicat.* 108:105680.

[B13] Elnahal A. S. M. El-Saadony M. T. Saad A. M. Desoky E.-S. M. El-Tahan A. M. Rady M. M. (2022). The use of microbial inoculants for biological control, plant growth promotion, and sustainable agriculture: A review. *Eur. J. Plant Pathol.* 162 759–792.

[B14] Fanin N. Kardol P. Farrell M. Nilsson M. C. Gundale M. J. Wardle D. A. (2019). The ratio of gram-positive to gram-negative bacterial PLFA markers as an indicator of carbon availability in organic soils. *Soil Biol. Biochem.* 128 111–114.

[B15] Griffiths B. S. Philippot L. (2013). Insights into the resistance and resilience of the soil microbial community. *FEMS Microbiol. Rev.* 37 112–129. 22568555 10.1111/j.1574-6976.2012.00343.x

[B16] Guo A. Zhao Z. Zhang P. Yang Q. Li Y. Wang G. (2019). Linkage between soil nutrient and microbial characteristic in an opencast mine. China. *Sci. Total Environ.* 671 905–913. 10.1016/j.scitotenv.2019.03.065 30947061

[B17] Jin L. Jin N. Wang S. Li J. Meng X. Xie Y. (2022). Changes in the microbial structure of the root soil and the yield of Chinese baby cabbage by chemical fertilizer reduction with bio-organic fertilizer application. *Microbiol. Spectr.* 10:e0121522. 10.1128/spectrum.01215-22 36377898 PMC9784769

[B18] Joergensen R. G. (2021). Phospholipid fatty acids in soil-drawbacks and future prospects. *Biol. Fertil. Soils* 58 1–6.

[B19] Kant S. (2018). Understanding nitrate uptake, signaling and remobilisation for improving plant nitrogen use efficiency. *Semin. Cell Dev. Biol.* 74 89–96. 28838687 10.1016/j.semcdb.2017.08.034

[B20] Khan S. T. (2022). Consortia-based microbial inoculants for sustaining agricultural activities. *Appl. Soil Ecol.* 176:104503. 10.1007/s00203-021-02446-9 34251477

[B21] Kumar C. M. Ghoshal N. (2017). Impact of land-use change on soil microbial community composition and organic carbon content in the dry tropics. *Pedosphere* 27 974–977.

[B22] Latifah O. Ahmed O. H. Majid N. M. A. (2017). Enhancing nitrogen availability from urea using clinoptilolite zeolite. *Geoderma* 306 152–159. 10.1177/0734242X15576771 25819928

[B23] Laurent C. Bravin M. N. Crouzet O. Pelosi C. Tillard E. Lecomte P. (2020). Increased soil pH and dissolved organic matter after a decade of organic fertilizer application mitigates copper and zinc availability despite contamination. *Sci. Total Environ.* 709:135927. 10.1016/j.scitotenv.2019.135927 31905571

[B24] Li J. Shao X. Huang D. Shang J. Liu K. Zhang Q. (2020). The addition of organic carbon and nitrogen accelerates the restoration of soil system of degraded alpine grassland in Qinghai-Tibet Plateau. *Ecol. Eng.* 158:106084.

[B25] Li J. Zhao Y. Shao X. Huang D. Shang J. Li H. (2021). The mixed addition of Biochar and nitrogen improves soil properties and microbial structure of moderate-severe degraded alpine grassland in qinghai-tibet plateau. *Front. Plant Sci.* 12:765041. 10.3389/fpls.2021.765041 34880889 PMC8647844

[B26] Li L. Zhang Y. Wu J. Li S. Zhang B. Zu J. (2019). Increasing sensitivity of alpine grasslands to climate variability along an elevational gradient on the Qinghai-Tibet Plateau. *Sci. Total Environ.* 678 21–29. 10.1016/j.scitotenv.2019.04.399 31075588

[B27] Li Y. Dong S. Liu S. Zhou H. Gao Q. Cao G. (2015). Seasonal changes of CO_2_, CH_4_ and N2O fluxes in different types of alpine grassland in the Qinghai-Tibetan plateau of China. *Soil Biol. Biochem.* 80 306–314.

[B28] Liang J. P. Xue Z. Q. Yang Z. Y. Chai Z. Niu J. P. Shi Z. Y. (2021). Effects of microbial organic fertilizers on *Astragalus membranaceus* growth and rhizosphere microbial community. *Ann. Microbiol.* 71:11.

[B29] Liao J. J. Dou Y. X. Yang X. An S. S. (2023). Soil microbial community and their functional genes during grassland restoration. *J. Environ. Manag.* 325: 116488. 10.1016/j.jenvman.2022.116488 36419280

[B30] Liu H. Zhang L. Sun Y. Xu G. Wang W. Piao R. (2021). Degradation of lignocelluloses in straw using AC-1, a thermophilic composite microbial system. *PeerJ* 9:e12364. 10.7717/peerj.12364 34760379 PMC8567851

[B31] Liu M. Yu C. Zhu T. Xu X. Wang Y. (2022). Restoration of degraded alpine grasslands alters plant–microbial competition for nitrogen. *Biol. Fertil. Soils* 58 803–814.

[B32] Liu Y. F. Fang H. Leite P. A. M. Liu Y. Zhao J. Wu G. L. (2023). Mattic epipedon fragmentation strengthened the soil infiltration capacity of a hillside alpine meadow on the Qinghai-Tibetan Plateau. *Ecohydrology* 16:e2552.

[B33] Luo Z. M. Liu J. X. He L. Du J. Q. Wang L. X. Jia T. (2022). Degradation-induced microbiome alterations may aggravate soil nutrient loss in subalpine meadows. *Land Degrad. Dev.* 33 2699–2712.

[B34] Lyu X. Li X. Dang D. Dou H. Wang K. Gong J. (2022). A perspective on the impact of grassland degradation on ecosystem services for the purpose of sustainable management. *Remote Sens.* 14:5120.

[B35] Mallon C. A. Poly F. Le Roux X. Marring I. van Elsas J. D. Salles J. F. (2015). Resource pulses can alleviate the biodiversity-invasion relationship in soil microbial communities. *Ecology* 96 915–926. 10.1890/14-1001.1 26230013

[B36] Megali L. Schlau B. Rasmann S. (2015). Soil microbial inoculation increases corn yield and insect attack. *Agron. Sust. Dev.* 35 1511–1519.

[B37] Mooshammer M. Wanek W. Hammerle I. Fuchslueger L. Hofhansl F. Knoltsch A. (2014). Adjustment of microbial nitrogen use efficiency to carbon: Nitrogen imbalances regulates soil nitrogen cycling. *Nat. Commun.* 5:3694. 10.1038/ncomms4694 24739236 PMC3997803

[B38] Nikolaou C. N. Chatziartemiou A. Tsiknia M. Karyda A. G. Ehaliotis C. Gasparatos D. (2023). Calcium- and magnesium-enriched organic fertilizer and plant growth-promoting Rhizobacteria affect soil nutrient availability, plant nutrient uptake, and secondary metabolite production in aloe vera (Aloe barbadensis Miller) grown under field conditions. *Agronomy* 13:482.

[B39] Niu B. Wang W. Yuan Z. Sederoff R. R. Sederoff H. Chiang V. L. (2020). Microbial interactions within multiple-strain biological control agents impact soil-borne plant disease. *Front. Microbiol.* 11:585404. 10.3389/fmicb.2020.585404 33162962 PMC7581727

[B40] Pan J. Peng Y. Wang J. Tian D. Zhang R. Li Y. (2023). Controlling factors for soil bacterial and fungal diversity and composition vary with vegetation types in alpine grasslands. *Appl. Soil Ecol.* 184:104777.

[B41] Peng F. Xue X. You Q. Sun J. Zhou J. Wang T. (2019). Change in the trade-off between aboveground and belowground biomass of alpine grassland: Implications for the land degradation process. *Land Degrad. Dev.* 31 105–117.

[B42] Ren Y. Lü Y. Fu B. (2016). Quantifying the impacts of grassland restoration on biodiversity and ecosystem services in China: A meta-analysis. *Ecol. Eng.* 95 542–550.

[B43] Rosinger C. Keiblinger K. M. Rousk J. Sandén H. (2022). Shifts in microbial stoichiometry upon nutrient addition do not capture growth-limiting nutrients for soil microorganisms in two subtropical soils. *Biogeochemistry* 159 33–43.

[B44] Rousk J. Brookes P. C. Baath E. (2009). Contrasting soil pH effects on fungal and bacterial growth suggest functional redundancy in carbon mineralization. *Appl. Environ. Microbiol.* 75 1589–1596. 19151179 10.1128/AEM.02775-08 PMC2655475

[B45] Rousk J. Brookes P. C. Bååth E. (2010). The microbial PLFA composition as affected by pH in an arable soil. *Soil Biol. Biochem.* 42 516–520.

[B46] Shi H. Lu L. Ye J. Shi L. (2022). Effects of two bacillus velezensis microbial inoculants on the growth and rhizosphere soil environment of *Prunus davidiana*. *Int. J. Mol. Sci.* 23:13639. 10.3390/ijms232113639 36362427 PMC9657632

[B47] Szymanek M. Dziwulska-Hunek A. Zarajczyk J. Michałek S. Tanaś W. (2020). The influence of red light (RL) and effective microorganism (EM) application on soil properties. Yield, and quality in wheat cultivation. *Agronomy* 10:1201.

[B48] Wang D. Zhou H. Zuo J. Chen P. She Y. Yao B. (2022). Responses of soil microbial metabolic activity and community structure to different degraded and restored grassland gradients of the Tibetan plateau. *Front. Plant Sci.* 13:770315. 10.3389/fpls.2022.770315 35463442 PMC9024238

[B49] Wang J. Li W. Cao W. Atio Abalori T. Liu Y. Xin Y. (2021). Soil bacterial community responses to short-term grazing exclusion in a degraded alpine shrubland – grassland ecotone. *Ecol. Indicat.* 130:108043.

[B50] White D. C. Davis W. M. Nickels J. S. King J. D. Bobbie R. J. (1979). Determination of the sedimentary microbial biomass by extractible lipid phosphate. *Oecologia* 40 51–62. 28309603 10.1007/BF00388810

[B51] Xiao X. Li J. Lyu J. Feng Z. Zhang G. Yang H. (2022). Chemical fertilizer reduction combined with bio-organic fertilizers increases cauliflower yield via regulation of soil biochemical properties and bacterial communities in Northwest China. *Front. Microbiol.* 13:922149. 10.3389/fmicb.2022.922149 35966650 PMC9363920

[B52] Yang C. Zhang H. Zhao X. Liu P. Wang L. Wang W. (2023). A functional metagenomics study of soil carbon and nitrogen degradation networks and limiting factors on the Tibetan plateau. *Front. Microb.* 14:1170806. 10.3389/fmicb.2023.1170806 37228377 PMC10203874

[B53] Yang W. S. Liu Y. Zhao J. Chang X. Wiesmeier M. Sun J. (2021). SOC changes were more sensitive in alpine grasslands than in temperate grasslands during grassland transformation in China: A meta-analysis. *J. Clean. Prod.* 308:127430.

[B54] Yu M. Zeng G. Chen Y. Yu H. Huang D. Tang L. (2009). Influence of Phanerochaete chrysosporium on microbial communities and lignocellulose degradation during solid-state fermentation of rice straw. *Process Bioch.* 44 17–22.

[B55] Zeng W. Wang Z. Chen X. Yao X. Wang W. (2021). Increased nitrogen availability alters soil carbon quality by regulating microbial r-K growth strategy, metabolic efficiency, and biomass in degraded temperate grasslands. *Land Degrad. Dev.* 32 3550–3560.

[B56] Zhang Y. Dong S. Gao Q. Liu S. Zhou H. Ganjurjav H. (2016). Climate change and human activities altered the diversity and composition of soil microbial community in alpine grasslands of the Qinghai-Tibetan Plateau. *Sci. Total Environ.* 562 353–363. 10.1016/j.scitotenv.2016.03.221 27100015

