# Supplementary material for: Effective microorganisms input efficiently improves the vegetation and microbial community of degraded alpine grassland
Source: Front Microbiol. 2024 Jan 15;14:1330149. doi: 10.3389/fmicb.2023.1330149 (PMC10829099; doi:10.3389/fmicb.2023.1330149)
Supplement: Supplementary file 1 [file Data_Sheet_1.docx]

**Table S1.** Microbial PLFA biomarkers and metrics

| Community Category | Community Metric | | PLFA Biomarker | | References |
| --- | --- | --- | --- | --- | --- |
| PLFA Biomass | Sum named and unnamed PLFAs | |  | |  |
| Total Bacterial PLFAs |  | | i14:0, 14:0, a15:0, i15:0, 15:0, i16:0, 16:0, 16:1w7, a17:0, i17:0, iso17:1, 17:0, 18:1 w5, 18:1 w7, 18:0, cy17:0 and cy19:0 | | Frostegard and Baath 1996;  Sundh et al. 2000;  Evgrafova et al. 2008;  Djukic et al. 2010;  Smith et al. 2015;  Li et al. 2020; |
| Actinomycetes PLFAs |  | | 10Me16:0, 10Me17:0 and 10Me18:0 | |  |
| Total Fungal PLFAs |  | | 18:1w9c,18:2w6,9c | |  |
| Gram-positive PLFAs |  | | i14:0, a15:0, i15:0, i16:0, i17:0, a17:0. | |  |
| Gram-positive PLFAs |  | | 16:1ω7, cy17:0, cy19:0 | |  |
| Indicator PLFAs | Gram-positive bacteria (Gp) | | 15:0iso | |  |
|  | Actinobacteria (Ab) | | 16:10 methyl | |  |
|  | Gram-negative bacteria (Gn) | | 16:1 w7c | |  |
|  | Arbuscular mycorrhizal fungi (Am) | | 16:1 w5c | |  |
|  | Saprotrophic fungi (Sf) | | 18:1 w9c, 18:2w6,9c | |  |
|  | Methanotrophic bacteria (Mb) | | 18:1 w7c | |  |
|  | | Anaerobic bacteria (An) | | 19:0 cyclo |  |

**References**

Djukic I, Zehetner F, Mentler A, Gerzabek MH (2010) Microbial community composition and activity in different Alpine vegetation zones. Soil Biol Biochem 42(2):155-161.

Evgrafova SY, Santruckova H, Shibistova OB, Elhottova D, Cerna B, Zrazhevskaya GK, Lloyd D (2008) Phospholipid fatty acid composition of microorganisms in pine forest soils of Central Siberia. Biol Bull 35(5):452-458.

Frostegard A, Baath E (1996) The use of phospholipid fatty acid analysis to estimate bacterial and fungal biomass in soil. Biol Fert Soils 22(1-2):59-65.

Li J, Shao X, Huang D, Shang J, Liu K, Zhang Q, Yang X, Li H, He Y (2020b) The addition of organic carbon and nitrogen accelerates the restoration of soil system of degraded alpine grassland in Qinghai-Tibet Plateau. Ecol Eng 158:106084.

Smith AP, Marin-Spiotta E, Balser T (2015) Successional and seasonal variations in soil and litter microbial community structure and function during tropical postagricultural forest regeneration: a multiyear study. Global Change Biol 21(9):3532-3547.

Sundh I, Borjesson G, Tunlid A (2000) Methane oxidation and phospholipid fatty acid composition in a podzolic soil profile. Soil Biol Biochem 32(7):1025-1028.


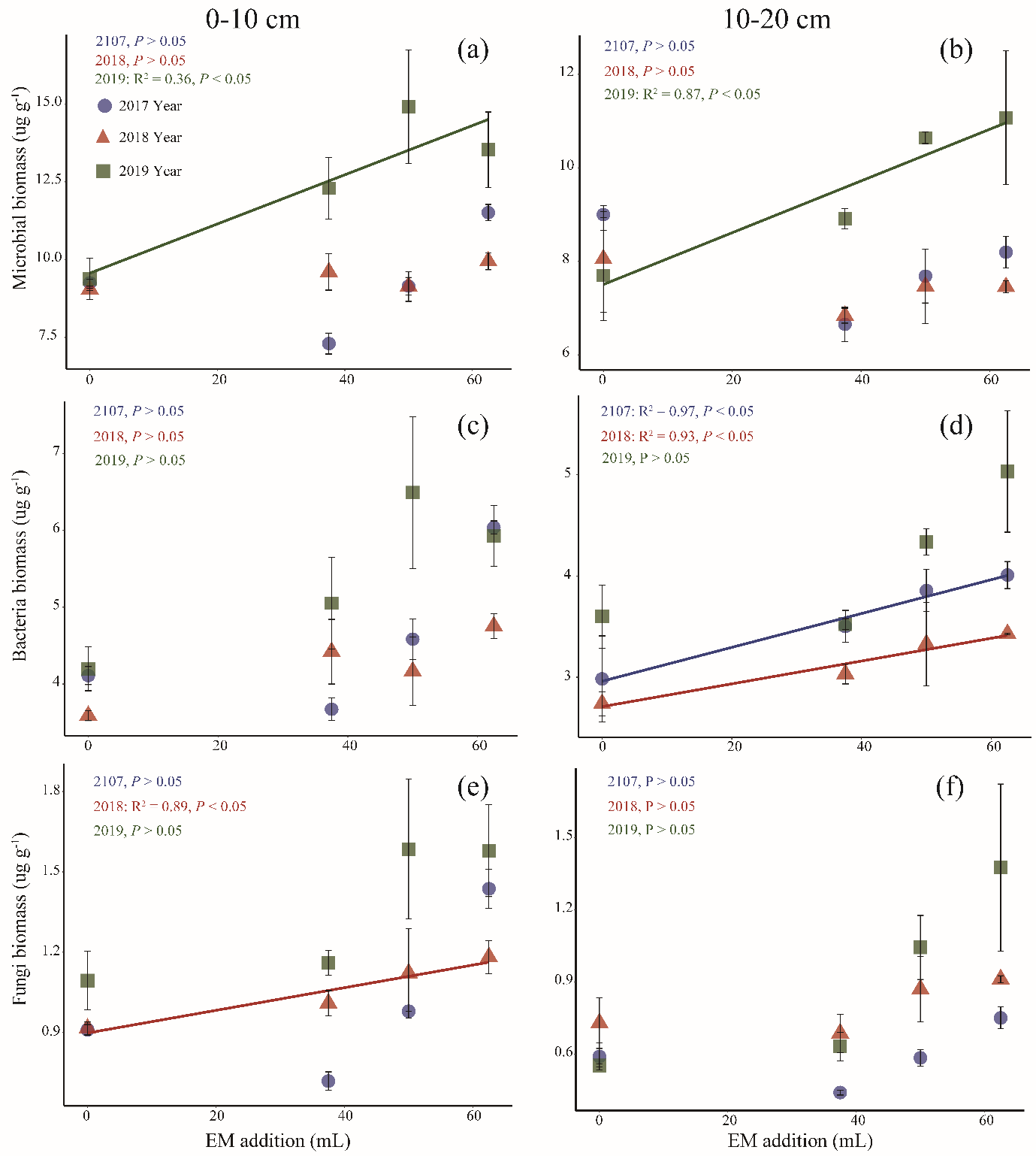


**Fig S1.** Effects of different EM addition levels on total microbial (a), bacterial (b) and fungal (c) biomasses in two soil layers. Values are means with standard errors (n = 3). Solid fitted lines are from the Pearson linear regression test.
